# Parasite Killing of* Leishmania (V) braziliensis* by Standardized Propolis Extracts

**DOI:** 10.1155/2017/6067172

**Published:** 2017-06-13

**Authors:** Jéssica Rebouças-Silva, Fabiana S. Celes, Jonilson Berlink Lima, Hernane S. Barud, Camila I. de Oliveira, Andresa A. Berretta, Valéria M. Borges

**Affiliations:** ^1^Instituto Gonçalo Moniz, Fundação Oswaldo Cruz, Salvador, BA, Brazil; ^2^Universidade Federal da Bahia, Salvador, BA, Brazil; ^3^Centro das Ciências Biológicas e da Saúde, Universidade Federal do Oeste da Bahia, Barreiras, BA, Brazil; ^4^Instituto de Química, Universidade Estadual Paulista, Araraquara, SP, Brazil; ^5^Programa de Pós-Graduação em Biotecnologia, Universidade de Araraquara, Araraquara, SP, Brazil; ^6^Apis Flora Industrial e Comercial Ltda., Laboratório de Pesquisa, Desenvolvimento e Inovação, Ribeirão Preto, SP, Brazil; ^7^Departamento de Ciências Farmacêuticas, Faculdade de Ciências Farmacêuticas de Ribeirão Preto, Universidade de São Paulo, Avenida do Café s/n, 14049-900 Ribeirão Preto, SP, Brazil

## Abstract

Treatments based on antimonials to cutaneous leishmaniasis (CL) entail a range of toxic side effects. Propolis, a natural compound widely used in traditional medical applications, exhibits a range of biological effects, including activity against infectious agents. The aim of this study was to test the potential leishmanicidal effects of different propolis extracts against* Leishmania (Viannia) braziliensis* promastigotes and intracellular amastigotes in vitro. Stationary-phase* L. (V) braziliensis *promastigotes were incubated with medium alone or treated with dry, alcoholic, or glycolic propolis extract (10, 50, or 100 *μ*g/mL) for 96 h. Our data showed that all extracts exhibited a dose-dependent effect on the viability of* L. (V) braziliensis* promastigotes, while controlling the parasite burden inside infected macrophages. Dry propolis extract significantly modified the inflammatory profile of murine macrophages by downmodulating TGF-*β* and IL-10 production, while upmodulating TNF-*α*. All three types of propolis extract were found to reduce nitric oxide and superoxide levels in activated* L*.* braziliensis*-infected macrophages. Altogether, our results showed that propolis extracts exhibited a leishmanicidal effect against both stages of* L. (V) braziliensis*. The low cell toxicity and efficient microbicidal effect of alcoholic or glycolic propolis extracts make them candidates to an additive treatment for cutaneous leishmaniasis.

## 1. Introduction

Leishmaniasis is a neglected vector-borne tropical disease caused by obligate protozoan parasites of the genus* Leishmania* [1–3]. Cutaneous leishmaniasis (CL) is the most common form of leishmaniasis worldwide, representing 50–75% of all new cases. According to the World Health Organization (WHO), the number of CL cases is around 1–1.5 million annually [4, 5]. In Brazil,* Leishmania (V) braziliensis* is the etiological agent mainly responsible for CL [6]. The CL lesion then ulcerates and may become secondarily infected with bacteria. Secondary bacterial infections in CL lesions are responsible for pain, can prolong disease duration, increase tissue destruction, and result in increased scarring [7, 8].

Treatment of CL can be complex, and this disease may be chronic and latent in the human host. Chemotherapy to treat leishmaniasis has been based on the parenteral administration of pentavalent antimonials for more than 60 years [9]. These compounds are highly toxic and expensive and have been associated with drug resistance [1]. Amphotericin B and Paromomycin, two other currently available second-line antileishmanial treatments [10, 11], also present significant shortcomings with regard to toxicity, cost, and duration of treatment. In this context, the search for new, safer, and more effective formulations [12], including substances from natural sources, offering less expensive and less toxic treatment options is urgently needed [13].

Propolis, a natural compound produced by* Apis mellifera* honeybees, has been widely used in traditional applications [14]. This substance has shown promising results against different infectious agents and exhibits a broad spectrum of biological properties [15–18]. The chemical composition of propolis is dependent on the biodiversity of each area visited by bees, as well as the method of extraction. These can influence the quantity and makeup of the specific biologically active compounds present in each sample, potentially leading to a range of biological effects [19, 20].

Previous studies using alcoholic and glycolic EPP-AF® extracts showed a potential antibacterial and antifungal effect in in vitro and in vivo models against* Staphylococcus aureus* (ATCC 25923),* Staphylococcus aureus* (ATCC 43300),* Staphylococcus epidermidis* (ATCC 14990) [21],* Saccharomyces cerevisiae* [22], and* Candida albicans *[23]. Berretta et al. [24] also showed the ability of glycolic extract to improve skin wound healing, such as in rat models of burn injury. Furthermore, a recent study also showed the ability of alcoholic propolis extract to inhibit the inflammasome, a key function mediated by the innate immune system, by reducing IL-1*β* secretion in mouse macrophages and decreasing the activation of caspase-1 [25].

Previous reports have shown that propolis extracts, mainly alcoholic extract, exhibit prominent microbicidal effects in vitro against* Leishmania* parasites, as well as reduced lesion size during experimental infection [26–30]. The present study shows for the first time a comparison of the potential leishmanicidal effect among distinct presentations of green propolis extracts, against promastigotes and intracellular amastigotes of* Leishmania (V.) braziliensis. *Uncovering which type of excipient and chemical presentation produces the extract with most potent leishmanicidal effect is critical for development of more effective adjunct therapies to patients with poor response to conventional antimicrobial treatment.

## 2. Methodology

### 2.1. Material and Reagents

Purified water (Milli-Q), HPLC grade methanol (J. T. Baker, L. 9093-68), formic acid (Vetec, L.0804789), caffeic acid (Fluka, L. 43706045),*ρ*-coumaric acid (Fluka, L.3250759), cinnamic acid (Fluka, L.21907066), isosakuranetin (ChromaDex), Artepillin C (Wako, L. 016.19131), 3,4-dicaffeoylquinic acid (Phytolab; L. 13672938; ≥90,0% de purity), 3,5-dicaffeoylquinic acid (Phytolab; L. 13672946; ≥90,0% of purity), 4,5-dicaffeoylquinic acid (Phytolab; L. 13672903; ≥90,0% purity), gallic acid (Synth, L.109250), sodium bicarbonate (Vetec, L.0906112), and aromadendrin-4′O-methyl ether were previously isolated, identified, and [31] kindly provided by the authors. High performance liquid chromatography (HPLC) was performed using a Shimadzu chromatograph equipped with a CBM-20A controller, LC-20AT quaternary pump, an SPD-M diodes 20A array detector, a Shimadzu Shim-Pack column CLC-ODS (M) (4.6 mm × 250 mm, 5 mm particle diameter, pore diameter, 100 Å), and Shimadzu LC software version 1.21 SP1. Schneider's insect medium, lipopolysaccharide (LPS), Acridine Orange, Ethidium Bromide, IFN-*γ*, and hydroxylamine were obtained from SIGMA-Aldrich (St Louis, MO, USA). Inactive fetal bovine serum (FBS), RPMI medium, penicillin, and Amphotericin B were purchased from GIBCO (Carlsbad, CA, USA). Streptomycin, L-glutamine, and Alamar Blue® were obtained from Invitrogen (Carlsbad, CA, USA). In addition, macrophage colony stimulating factor (M-CSF) was purchased from PEPROTECH (Rocky Hill, NJ, USA), mouse TNF-alpha and TGF-beta 1 Quantikine ELISA kits were obtained from R&D Systems (Minneapolis, MN), and a superoxide dismutase activity assay kit was purchased from Cayman Chemical Company (Ann Arbor, Michigan).

### 2.2. Propolis Extract Preparation

Standardized alcoholic propolis extract (Batch 1402110) (PSE), glycolic propolis extract (Batch 1480210) (PGE), and water-soluble propolis dry extract (Batch 9050213) (PSDE) were produced by Apis Flora (Ribeirão Preto/SP-Brazil) by formulating a mixture of raw propolis materials [24]. These “blends” of raw propolis material were made to effectively standardize the qualitative and quantitative chemical composition of all batches, that is, ensuring reproducibility, since the compounds and concentrations of substances present in propolis vary in accordance with each geographical region of production (the states of Minas Gerais, São Paulo, Paraná, Santa Catarina, and Rio Grande do Sul) [24].

To prepare the three different extracts, a mixture of raw propolis materials was first kept in a freezer for 12 hours and then reduced to a fine powder under maceration. To obtain each type of extract, standardized crude propolis material was initially extracted using an alcohol solution (7 : 3) in a dynamic maceration process, followed by percolation and filtration. Propolis glycolic extract (PGE) was obtained from standardized alcoholic propolis extract after evaporation of the ethanol portion and the addition of propylene glycol. The alcoholic and glycolic extracts contained 11% w/v of propolis dry matter. The dry propolis extract was obtained via a concentration of alcoholic extract containing around 80% of propolis dry material, followed by alkaline hydrolysis and conversion in aqueous extract by slowly adding heated purified water. The hydrolysis process following the evaporation of the hydroalcoholic solvent resulted in the ionization of the compounds found in propolis, transforming them into water-soluble structures (aqueous extract) [32]. Maltodextrin, at a ratio of 7 : 3 (propolis : maltodextrin by dry weight), was added and mixed under stirring and then is dried using a spray drying process.

### 2.3. HPLC Chemical Characterization

The propolis extracts were quantitatively analyzed on high performance liquid chromatography (HPLC). The mobile phase consisted of a gradient of methanol and acidified water with formic acid (0.1% v/v) ranging from 20% to 95%, for a runtime of 77 minutes at a flow rate of 0.8 mL/min. The detection wavelength was set at 275 nm [24]. To assess the flavonoid content in the extracts, the aluminium chloride method was employed as previously described by Woiskya and Salantino [33]. All samples were prepared in accordance with the mass and dilutions necessary to quantification in the analytical curve.

### 2.4. Biological Assay

#### 2.4.1. Ethics Statement

Male BALB/c mice aged 6–8 weeks were obtained from the animal care facility at CPqGM/FIOCRUZ, located in the city of Salvador, Bahia, Brazil. All animal experimentation was conducted in accordance with the Guidelines for Animal Experimentation as established by the Brazilian Council for Animal Experimentation Control (CONCEA). The present study received approval from the local institutional review board (CEUA) (protocol: CEUA-015/2015-CPqGM/FIOCRUZ).

#### 2.4.2. Parasites

*Leishmania Viannia braziliensis* (MHOM/BR/01/BA788) parasites were cultured in Schneider's insect medium supplemented with 10% inactive fetal bovine serum (FBS), 100 U/mL penicillin, 100 mg/mL streptomycin, and 2 mM L-glutamine in 25 cm^2^ flasks at 24°C for seven days.

#### 2.4.3. *L. (V.) braziliensis* Promastigote Viability Assay

Stationary-phase* L. (V.) braziliensis* promastigotes (2 × 10^5^/mL) were cultivated in supplemented Schneider medium (as described above) alone or in the presence of three concentrations (10, 50, and 100 *μ*g/mL) of dry, alcoholic, or glycolic propolis extract. Amphotericin B (0.25 *μ*g/mL) was used as a positive control. All cultures were incubated for 120 hours at 24°C, after which the number of viable promastigotes was determined by direct counting performed daily in a Neubauer Chamber.

#### 2.4.4. Fluorescence Microscopy

Stationary-phase* L. (V.) braziliensis* promastigotes (5 × 10^5^/mL) were cultured in supplemented Schneider medium (as described above) alone or in the presence of dry, alcoholic, and glycolic propolis extract (50 *μ*g/mL) for 96 h at 24°C. Treatment with Amphotericin B (0.5 *μ*g/mL) was used as positive control. The samples were mounted on cytospin slides to fluorescence microscopy. A solution containing 100 *μ*g/mL Acridine Orange and 100 *μ*g/mL Ethidium Bromide was prepared as previously described and added to the slides containing promastigotes [34, 35]. Parasite staining was assessed using a fluorescence microscope (OLYMPUS, Japan). Parasite promastigotes were considered alive when positively stained by Acridine Orange, while Ethidium Bromide staining was used to detect dead cells.

#### 2.4.5. Scanning Electron Microscopy

Stationary-phase* L. (V.) braziliensis* promastigotes (5 × 10^5^/mL) were cultured in supplemented Schneider medium (as described above) with dry, alcoholic, and glycolic propolis extracts (50 *μ*g/mL) for 96 h at 24°C. Supplemented Schneider medium was used as a control. The samples were then attached to coverslips and fixed in a solution containing 2.5% glutaraldehyde and 2% paraformaldehyde in 0.1 M sodium cacodylate buffer (pH = 7.4). After fixation, cells were washed in cacodylate buffer and postfixed with 1% osmium tetroxide. All samples were then dehydrated in an ethanol series (70, 80, 90, and 100°GL). Cells were finally dried by the critical point method, mounted on stubs, coated with gold (20–30 nm), and observed in a Jeol JSM 6390LV scanning electron microscope.

#### 2.4.6. Transmission Electron Microscopy

Stationary-phase* L. (V.) braziliensis* promastigotes (10^7^/mL) were cultured in Schneider's medium with dry extract and alcoholic and glycolic propolis extracts (50 *μ*g/mL) for 96 h. Supplemented Schneider medium was used as a control. All samples were fixed and postfixed as described above. Cells were then dehydrated in an acetone series (70, 80, 90, and 100°GL) and embedded in Polybed resin. Ultrathin sections were mounted on 300-mesh grids, stained with 5% uranyl acetate and lead citrate, and then observed using a Jeol Jem 1230 transmission electron microscope.

#### 2.4.7. Macrophage Toxicity Assay

Human macrophages (3 × 10^5^/well) were isolated from peripheral blood mononuclear cells (PBMC) of healthy donors by Ficoll gradient centrifugation and plastic adherence and then allowed to differentiate into macrophages in vitro (7 days), with RPMI medium supplemented with 10% FBS, 100 U/mL penicillin, 100 mmg/mL streptomycin, and 2 mM L-glutamine and 50 nM of M-CSF.

Bone marrow-derived murine (BMM) cells were harvested from BALB/c mice femurs and cultured at 37°C under 5% CO_2_ for 7 days in RPMI medium supplemented with 20% FBS, 100 U/mL penicillin, 100 mg/mL streptomycin, and 2 mM L-glutamine and 30% L929 cell culture supernatant as a source of macrophage colony stimulating factor. Next, differentiated BMMs were detached from the plate using cold saline solution. BMMs (10^5^/well) were plated in 96-well plates and cultured at 37°C under 5% CO_2_ in RPMI-supplemented medium for 24 hours.

Human and BMMs uninfected macrophages were then treated with either dry extract and alcoholic or glycolic propolis extracts at varying concentrations (10, 50, and 100 *μ*g/mL) at 37°C for 48 h. Next, the cells were reincubated for another 4 h with supplemented RPMI medium containing 10% Alamar Blue. The reagent absorbance was read at 570 nm and 600 nm using a spectrophotometer (SPECTRA Max 190). Hydrogen peroxide (H_2_O_2_) was used as positive control.

#### 2.4.8. Macrophage Infection

Human and BMM monocytes were isolated as described above and 2 × 10^5^/cells per well were seeded in 96-well plates. Macrophages were infected (10 : 1) with stationary-phase* Leishmania (V.) braziliensis* (MHOM/BR/01/BA788) promastigotes for 24 h and treated with varying concentrations (10, 50, and 100 *μ*g/mL) of one of the three propolis extracts for 48 h. Next, the medium was replaced with 0.2 mL of supplemented Schneider medium. Cells were then cultured at 24°C for an additional five days and the number of viable parasites was determined by direct counting. Amphotericin B (0.25 *μ*g/mL) was used as positive control.

#### 2.4.9. Quantification of Inflammatory and Oxidative Stress Mediators

BMMs (10^6^/well) were stimulated with IFN-*γ* (100 UI/mL) for 24 h and infected with* L. (V) braziliensis* stationary-phase promastigotes (10^7^/well) for another 24 h. The macrophages were then washed to remove any noninternalized parasites, the RPMI cell medium was replaced, and IFN-*γ* stimulation was reapplied together with 50 *μ*g/mL of dry extract and alcoholic or glycolic propolis extract for 48 h [13, 36]. Next, culture supernatants were collected. The Griess reaction was used to measure nitric oxide (NO) and superoxide (O_2_^−^) production. O_2_^−^ production was assessed by adding hydroxylamine (0.5 mM) to infected macrophages, which converts superoxide into nitrite [37]. Background levels of nitrite generated by the release of NO were determined in parallel with O_2_^−^, without the addition of hydroxylamine [13]. Superoxide dismutase (SOD) activity was determined using an SOD activity assay kit. Production of IL-10, TGF-*β*, and TNF-*α* was evaluated using a Quantikine ELISA kit in accordance with manufacturer instructions.

#### 2.4.10. Statistical and Data Analyses

Data are presented as the mean ± standard deviation (SD) from experiments performed in quintuplicate. GraphPad Prism Software 5.0 (GraphPad, San Diego, CA) was used for all data analyses. The Kruskal–Wallis nonparametric test with Dunn's posttest was used for multiple comparisons. Linear trend ad hoc analysis was used to evaluate statistical significance among the groups, considered when *p* < 0.05.

## 3. Results

### 3.1. Chemical Characterization of Standardized Propolis Extracts

The chemical compounds found in each type of extract, following normalization to 11% of propolis dry matter, are listed in Table 1. Alcoholic extract presented higher values for each compound, except Artepillin C, baccharin, and total flavonoids as quercetin (5.329, 0.500, and 5.794 mg/g resp.). Glycolic extract showed the highest values of 3,5-dicaffeoylquinic acid (1.703 mg/g) and total flavonoids (6.625 mg/g), while dry extract (dryness) showed markedly more caffeic acid (0.642 mg/g), Artepillin C (7.076 mg/g), and baccharin (0.907 mg/g).

### 3.2. Exposure to Propolis Extract Reduces the Viability of* Leishmania (V.) braziliensis* Promastigotes

**Figure 1:**
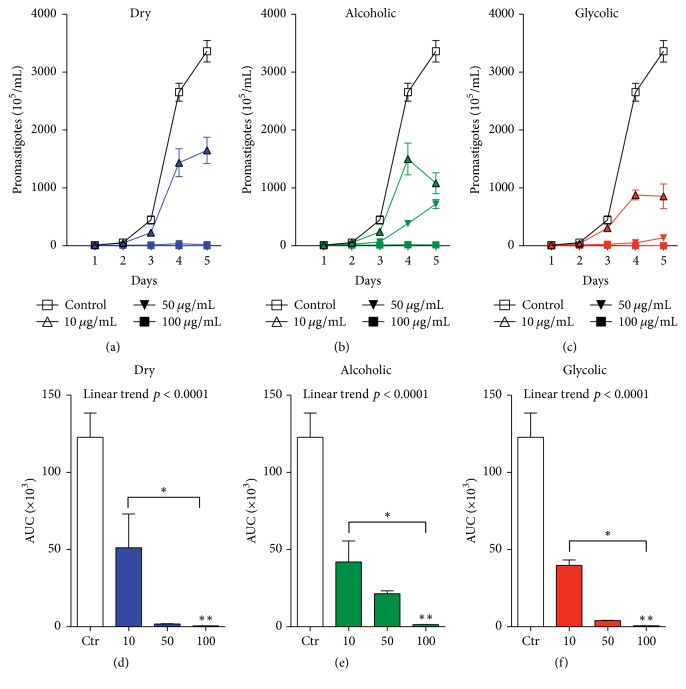
Dose-response effect of propolis extract on* L (V). braziliensis* promastigote viability. Parasites were incubated for 96 h with 10, 50, or 100 ug/mL of either (a and d) dry, (b and e) alcoholic, or (c and f) glycolic propolis extract. Bars represent means ± SD of two representative experiments performed in quadruplicate. AUC accounts for Area under the Curve and it corresponds to the area of the geometric figure made by the concentration curve of a drug as a function of time. The Kruskal–Wallis nonparametric test, followed by Dunn's posttest, was used to compare among experimental groups (^*∗*^*p* < 0.05, ^*∗∗*^*p* < 0.01, and, posttest for linear trend, *p* < 0.0001).

**Figure 2:**
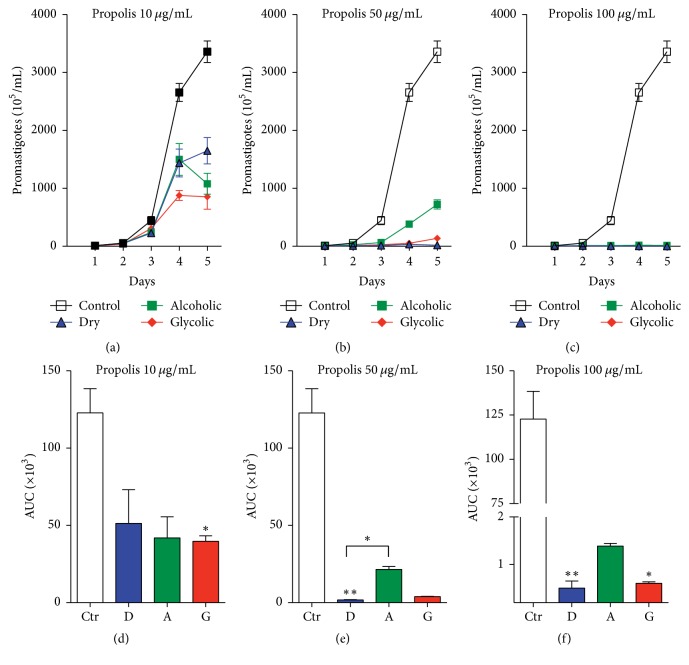
Viability of* L. (V) braziliensis *promastigotes in response to each type of propolis extract. Parasites were incubated for 96 h with dry, alcoholic, and glycolic propolis extract at (a and d) 10 *μ*g/mL, (b and e) 50 *μ*g/mL, or (c and f) 100 *μ*g/mL. Bars represent means ± SD of two representative experiments performed in quadruplicate. AUC accounts for Area under the Curve and it corresponds to the area of the geometric figure made by the concentration curve of a drug as a function of time. The Kruskal–Wallis nonparametric test, followed by Dunn's posttest, was used to compare among experimental groups (^*∗*^*p* < 0.05, ^*∗∗*^*p* < 0.01, and, posttest for linear trend, *p* < 0.0001).

To analyze the direct effect of each type of propolis extract on parasite viability, stationary-phase* L. (V.) braziliensis* promastigotes were incubated with one of three extracts: dry, alcoholic, and glycolic extract at different concentrations (10, 50, and 100 *μ*g/mL) for 96 h. The viability assay (Figures 1(a)–1(c)) showed a dose-dependent reduction in the number of viable promastigotes in comparison with untreated controls, as demonstrated by analysis of the Area under the Curve (Figures 1(d)–1(f)). The propolis extracts used at 50 *μ*g/mL and 100 *μ*g/mL concentrations demonstrated significant leishmanicidal effect in comparison to untreated controls (Figure 2). The following reductions in promastigotes were observed at treatment concentrations of 10, 50, and 100 *μ*g/mL, respectively, in comparison to untreated parasites (123 ± 15.6): dry extract: 58.3% (58.3 ± 21.8), 98.5% (1.7 ± 0.19), and 99.5% (0.5 ± 0.14); alcoholic extract: 65.9% (41.9 ± 13.6), 82.6% (21.4 ± 1.8), and 98.8% (1.3 ± 0.05); glycolic extract: 67.7% (39.7 ± 3.5), 96.7% (3.96 ± 0.16), and 99.5% (0.6 ± 0.03). Treatment with Amphotericin B (0.25 *μ*g/mL), used as positive control for leishmanicidal activity, completely eliminated all parasites after 24 h of treatment (data not shown).

**Figure 3:**
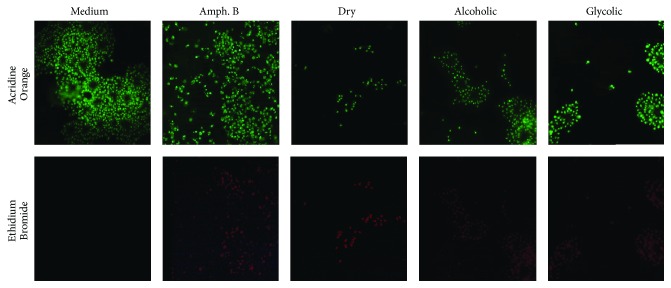
*L*.* (V) braziliensis* promastigote viability assessed by fluorescence microscopy. Live cells were stained with Acridine Orange (green color) and dying cells with Ethidium Bromide (red color).* L. braziliensis* promastigotes were treated with medium alone or with dry, alcoholic, or glycolic extracts of propolis (50 *μ*g/mL) for 96 h. Amphotericin B treatment (0.5 *μ*g/mL) for 24 h was used as a positive control for cell death. Magnification: 1000x.

The propolis extract treatment concentration was standardized at 50 *μ*g/mL for subsequent promastigote experimentation and analysis. Moreover, fluorescence microscopy employing double staining with Acridine Orange/Bromide Ethidium (OA/BE) (Figure 3) revealed that no significant cell death was observed in the untreated control group (medium), whereas all types of propolis extract (50 *μ*g/mL), as well as the positive control (Amph. B), successfully killed* L. (V) braziliensis* in vitro (Figure 3).

### 3.3. Morphological and Ultrastructural Analyses of Treated* Leishmania (V.) braziliensis* Promastigotes

**Figure 4:**
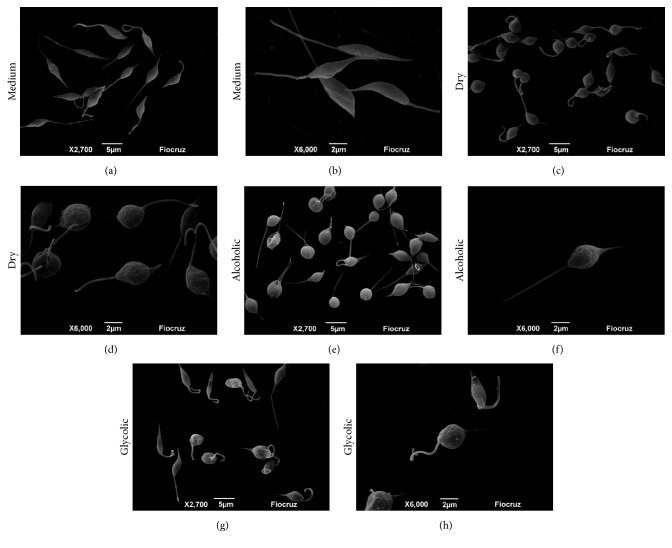
Scanning electron microscopy of morphological changes in* L*.* (V) braziliensis* promastigotes. Parasites were treated with either medium alone or dry, alcoholic, or glycolic extracts of propolis (50 *μ*g/mL) for 96 h. Image amplifications show scale bar representative of 5 *μ*m or 2 *μ*m.

Scanning electron microscopy (SEM) was employed to document morphological changes induced by treatment with propolis extract. Normal morphology was preserved in untreated parasites (Figures 4(a) and 4(b)), for example, stable cell surfaces, typically elongated shapes, and longer flagella. On the other hand, parasites treated with each type of propolis extract (50 *μ*g/mL) exhibited marked morphological changes, such as cell shrinkage and rounded forms, in addition to irregular surface protrusions and indentations in the plasma membrane (Figures 4(c)–4(h)).

**Figure 5:**
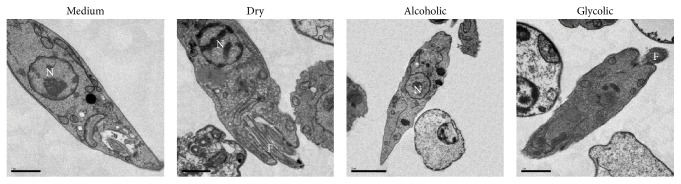
Transmission electron microscopy of ultrastructural changes and cell death in* L*.* (V) braziliensis* promastigotes. Parasites were treated with either medium alone or dry, alcoholic, or glycolic extracts of propolis (50 *μ*g/mL) for 96 h. Nucleus (N). Flagellum (F). Scale bars represent 1 *μ*m.

Transmission electron microscopy (TEM) was used to investigate ultrastructural changes in treated* L. (V) braziliensis* promastigotes (Figure 5). Analysis by TEM showed that control parasites retained normal morphology, with clearly defined membranes, preserved nuclei, Golgi complex, and flagellar pocket. In contrast, the parasites treated with alcoholic and glycolic extracts showed electron-dense granules in the cytoplasm without well-demarcated cytoplasmic organelles, suggestive of cell death. The parasites treated with dry extract showed less-marked morphological alterations, with irregular cell surfaces and an increased density of electron-dense granules in comparison to untreated promastigotes.

### 3.4. Macrophage Viability and the Leishmanicidal Effects of Propolis Extract on Human and Murine Macrophages In Vitro

**Figure 6:**
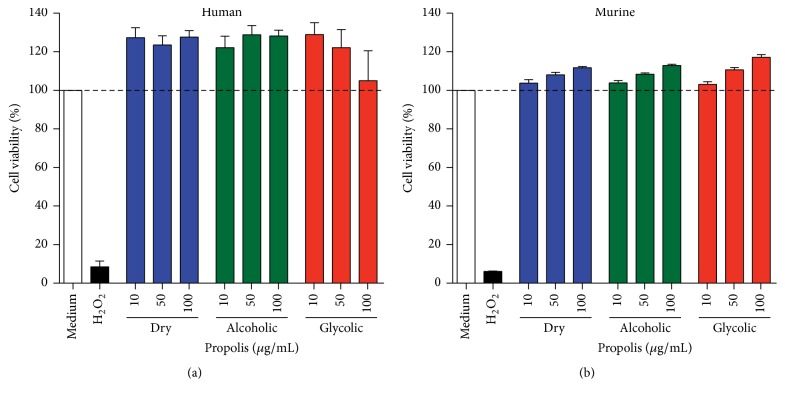
Cell cytotoxicity assessment by Alamar Blue. Data representative of viability of uninfected (a) human and (b) murine macrophages treated for 48 h with either medium alone or the dry, alcoholic, or glycolic (10, 50, and 100 *μ*g/mL) extracts of propolis. Hydrogen peroxide (H_2_O_2_) was used as a positive control for cell death. Bars represent mean ± SD values of three representative experiments performed using cells from six healthy human donors or in quintuplicate for murine cells.

Cell viability was unaffected by each concentration propolis extract tested, as assayed by Alamar Blue, in human (Figure 6(a)) and BALB/c murine macrophages (Figure 6(b)).

**Figure 7:**
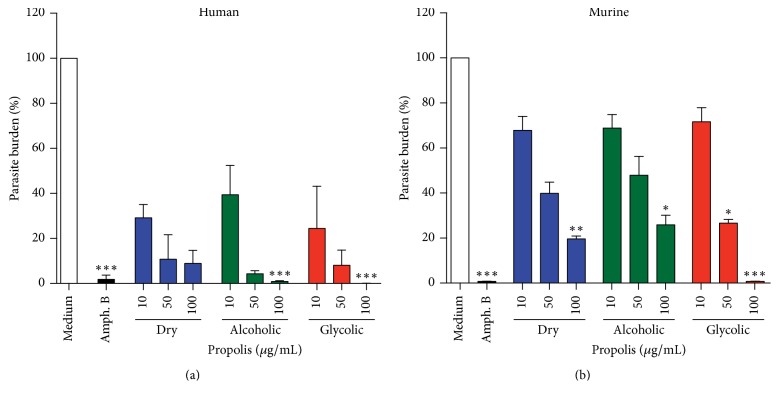
Evaluation of viability of promastigotes recovered from* L. (V) braziliensis*-infected macrophages. Human (a) and murine (b) macrophages infected for 24 h with* L*.* braziliensis, *treated with medium alone or with dry, alcoholic, or glycolic extracts of propolis (10, 50, and 100 *μ*g/mL) for 48 h. Amphotericin B (0.25 *μ*g/mL) was used as a positive control. Bars represent means ± SD of two representative experiments performed using cells from six healthy human donors or in quintuplicate for murine cells. Kruskal–Wallis nonparametric test, followed by Dunn's posttest, was used to compare among experimental groups (^*∗*^*p* < 0.05, ^*∗∗*^*p* < 0.01, and ^*∗∗∗*^*p* < 0.001).

The leishmanicidal effect of varying concentrations (10, 50, and 100 *μ*g/mL) of the three propolis extracts (dry, alcoholic, and glycolic extract) was evaluated in human and BALB/c macrophages infected with* L. (V.) braziliensis* (Figure 7). A significant reduction in the number of viable promastigotes recovered from infected cells was observed in human macrophages treated with alcoholic or glycolic extract, in comparison to untreated cells. The propolis extract treatment concentration was standardized at 50 *μ*g/mL for all subsequent macrophage experimentation and analysis.

### 3.5. Inflammatory Mediator Production in Response to Propolis Extract Treatment in* L. (V) braziliensis*-Infected Murine Macrophages

**Figure 8:**
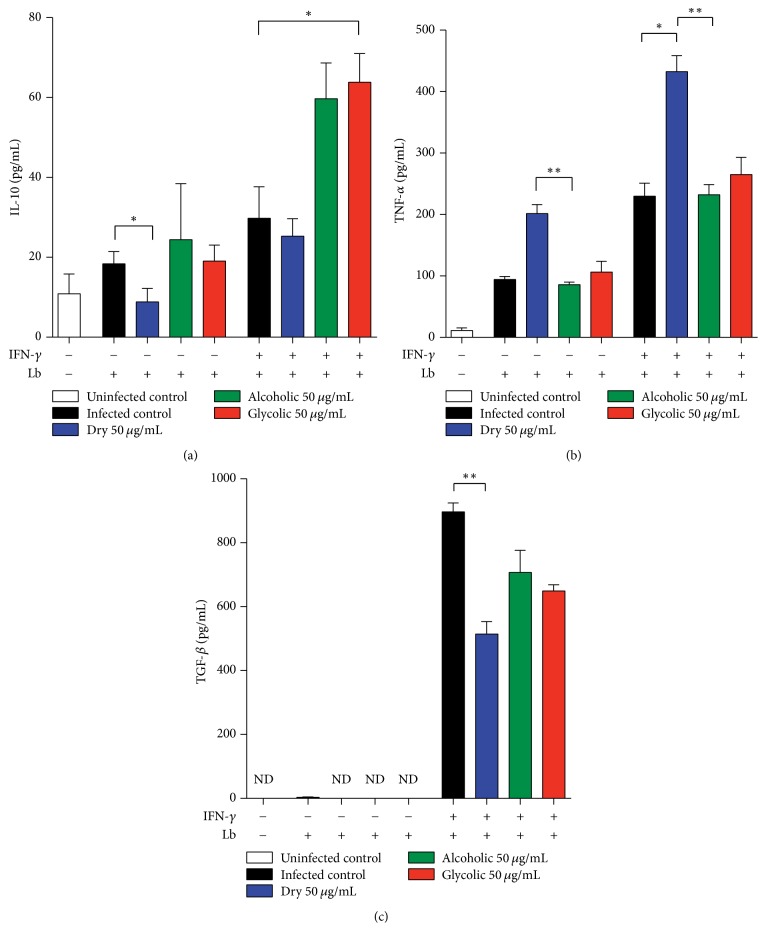
Modulation of cytokine production by propolis extract treatment. Quantification of inflammatory mediators secreted by propolis extract-treated* L. (V) braziliensis*-infected macrophages, stimulated by IFN-*γ* or not, and measured in cellular supernatant, as described in Materials and Methods: (a) interleukin-10 (IL-10), (b) Transforming Growth Factor (TGF-*β*), and (c) tumor necrosis factor (TNF-*α*). The Kruskal–Wallis nonparametric test, followed by Dunn's posttest, was used to compare among experimental groups (^*∗*^*p* < 0.05 and ^*∗∗*^*p* < 0.01).

Cytokine production by unstimulated, or INF-*γ*-activated, infected murine macrophages was assessed in vitro (Figure 8). Similar levels of IL-10 and TNF-*α* were seen in unstimulated infected macrophages treated with alcoholic and glycolic extracts, without statistical significance in relation to untreated infected cells. In contrast, the unstimulated infected macrophages treated with dry extract exhibited downmodulated IL-10 but upregulated TNF-*α* production, when compared to untreated infected cells. Additionally, increased IL-10 production was observed in the INF-*γ*-activated* L. (V) braziliensis*-infected macrophages treated with glycolic extract in comparison to activated infected controls. Finally, dry extract was observed to downmodulate TGF-*β*, but upregulate TNF-*α* production, in INF-*γ*-activated infected macrophages, compared to activated infected controls (Figure 8).

### 3.6. Antioxidant Effects of Propolis Extract in* L. (V) braziliensis*-Infected Murine Macrophages

**Figure 9:**
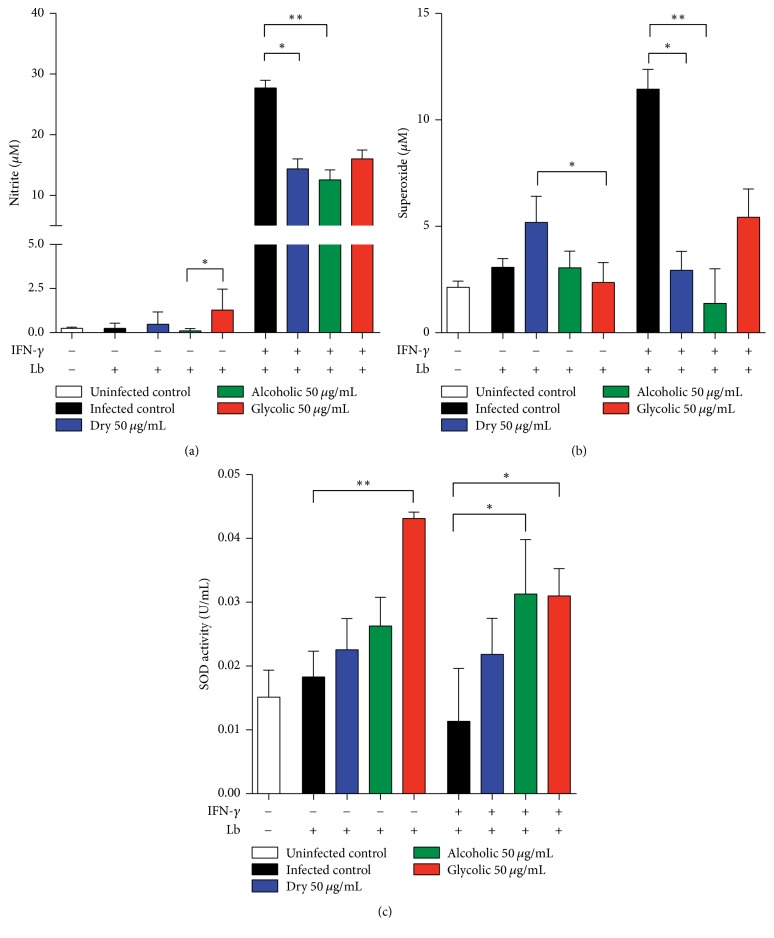
Modulation of oxidative response by propolis extract treatment. Quantification of mediators of oxidative stress released by propolis extract-treated* (V) braziliensis*-infected macrophages, stimulated by IFN-*γ* or not, and measured in cellular supernatant, as described in Materials and Methods: (a) nitric oxide; (b) superoxide; (c) superoxide dismutase (SOD) activity. The Kruskal–Wallis nonparametric test, followed by Dunn's posttest, was used to compare among experimental groups (^*∗*^*p* < 0.05 and ^*∗∗*^*p* < 0.01).

All three types of propolis extract were found to reduce nitric oxide and superoxide levels in activated* L. (V) braziliensis*-infected macrophages (Figure 9). Moreover, glycolic extract was observed to significantly increase SOD activity in both nonactivated and activated* L. (V) braziliensis*-infected macrophages, while alcoholic extract significantly increased SOD activity only in activated* L. (V) braziliensis*-infected macrophages, all in comparison to INF-*γ*-activated infected macrophages.

## 4. Discussion

Propolis, a complex and resinous substance composed of variable vegetable sources and honeybee secretions [20, 38], has a broad spectrum of biological effects and, therefore, has attracted the attention of scientists as an alternative to traditional treatment in several diseases [15, 39]. Several studies have shown antileishmanial effects in vitro and in vivo in* Leishmania* parasites using murine models employing propolis extracts from different sources, including propolis from Paraná, red propolis from Alagoas, and green propolis from Minas Gerais in Brazil [14, 26, 27, 40–43].

The chemical composition of propolis varies greatly in accordance with the climatic conditions and local flora at the site of collection [15, 28–30], in addition to the method of extraction. Here, we used standardized green propolis presented in three different pharmaceutical preparations: dry, alcoholic, and glycolic extract. The most prevalent chemical components detected were phenolic acids (e.g., p-coumaric acid, drupanin, Artepillin C, caffeic acid, and baccharin), which is consistent with previous reports [21, 44–46]. Moreover, Artepillin C, a characteristic compound found in Brazilian green propolis, was the primary component found in the propolis extracts utilized herein. Phenolic compounds have been associated with antimicrobial, antioxidant, trypanocidal, and antitumoral activities [44, 47–52]. Previous studies have demonstrated the antileishmanial effects of* p*-coumaric acid and quercetin in in vitro and in vivo models [53, 54]. Herein, the levels of* p*-coumaric acid were found to be similar in all extracts evaluated, while total flavonoid content was higher for glycolic and alcoholic extracts (Table 1). Although it is extremely important to elucidate the chemical composition of propolis, its distinct pharmacological activities are considered complex and may be the result of synergistic interactions among various chemical compounds [55].

All propolis extracts demonstrated a direct effect against the proliferation of axenic promastigotes. The dosage of 50 *μ*g/mL was shown to reduce parasite viability, verified by fluorescent double staining with Acridine Orange and Ethidium Bromide. Furthermore, the alcoholic and glycolic extracts were also shown to induce morphological and ultrastructural changes in parasites, whereas dry extract was only able to induce morphological changes. Similar morphological and ultrastructural changes consistent with decreased cell viability have been previously reported, suggestive of an apoptosis-like process induced by drugs traditionally used to treat leishmaniasis [56, 57].

None of the three propolis extracts exhibited any cytotoxic effects on BMMs or human cells after 48 h of treatment, as determined by the Alamar Blue assay. Importantly, in in vitro infection, all propolis extracts were shown to reduce* L. (V) braziliensis *burden in a dose-dependent manner. This finding is in agreement with previous reports that obtained propolis from different sources [19, 41, 58]. Moreover, treatment with alcoholic and glycolic extracts was shown to be more effective at reducing parasite numbers than dry extract, perhaps due to the distinct chemical compositions among these extracts.

The* L. (V) braziliensis*-infected macrophages treated with propolis extract were shown to modulate the production of inflammatory mediators, as well as markers of oxidative stress. Dry propolis extract significantly modified the inflammatory profile of murine macrophages by downmodulating TGF-*β* and IL-10 production, while upmodulating TNF-*α*. TGF-*β* and IL-10 are known to play an important role in macrophage deactivation, leading to increased parasite load in experimental models of* L. (L) amazonensis*,* L. (V) braziliensis*, and* L. (L) major* infection [59–62]. In addition, dos Santos Thomazelli et al. [63] reported an immunomodulatory effect by treatment with hydroalcoholic propolis extract on PBMCs from CL patients, as well as on PBMCs from healthy donors infected or not with* Leishmania (V) braziliensis*, in which the extract was shown to increase IL-4 and IL-17, while decreasing IL-10 levels.

The generation of reactive oxygen species (ROS), especially superoxide, and tumor necrosis factor-alpha (TNF-*α*) had both been shown to play an important role in the control of cutaneous leishmaniasis. Previous reports have demonstrated that increases in TNF-*α* and superoxide production effectively decrease parasite load, resulting in the elimination of* L (V). braziliensis* in vitro [64]. All of the extracts herein confirmed the antioxidant effects of propolis, as evidenced by significant decreases in nitric oxide and superoxide production [65–69]. In addition, our results showed increased superoxide dismutase (SOD) activity following treatment by alcoholic and glycolic extracts. Pronounced SOD-1 expression levels were also detected in biopsies from New World cutaneous leishmaniasis patients [70]. The results presented herein demonstrate that different presentations of propolis extract reacted differently against* L. (V) braziliensis*. Numbers of intracellular amastigotes were reduced following treatment of infected host cells with dry extract, which was shown to produce greater levels of TNF-*α* than murine macrophages treated with alcoholic and glycolic propolis extract. This is suggestive of host cell activation, which may have led to parasite killing via the TNF-*α* pathway. Furthermore, host cells treated with the alcoholic and glycolic extracts both were shown to effectively reduce the number of amastigotes, similarly to the dry extract, but with significant increases in SOD, which is indicative of the downmodulation of the oxidative stress response. Further investigations are warranted to elucidate the underlying mechanisms by which the alcoholic and glycolic extracts act upon specific pathways, potentially offering both control of parasite burden and an anti-inflammatory effect.

## 5. Conclusions

Our findings demonstrate that propolis induced-inflammatory imbalance involving cytokines and oxidative response hallmarks the outcome of* Leishmania* infection. This would be extremely beneficial in the context of CL treatment, due to the high inflammatory profile of this disease, which leads to severe tissue damage.

## Figures and Tables

**Table 1:** Chemical characterization of propolis samples.

Compounds	Alcoholic (mg/g)	Glycolic (mg/g)	Dry (mg/g)
Caffeic acid	0.215 ± 0.001	0.200 ± 0.001	0,642 ± 0.032
p-Coumaric acid	1.315 ± 0.008	1.272 ± 0.006	1.037 ± 0.003
3,5-Dicaffeoylquinic acid	1.617 ± 0.020	1.703 ± 0.017	0.696 ± 0.019
4,5-Dicaffeoylquinic acid	3.732 ± 0.231	2.488 ± 0.031	1.422 ± 0.012
Cinnamic acid	0.306 ± 0.014	ND	0.029 ± 0.001
Aromadendrin	0.955 ± 0.028	0.479 ± 0.027	0.064 ± 0.007
Drupanin	3.254 ± 0.060	2,993 ± 0.148	2.167 ± 0.008
Artepillin C	5.329 ± 0.077	4.675 ± 0.182	7.076 ± 0.040
Baccharin	0.500 ± 0.026	0.452 ± 0.045	0.907 ± 0.007
Total flavonoids	5.794 ± 0.017	6.625 ± 0.026	4,744 ± 0.359

Alcoholic and glycolic extract contained 11% w/v of propolis dry matter (*n* = 3). Data shown represent mean and SD values. ND: not detected.
